# Supplementary material for: Is the Proteome of Bronchoalveolar Lavage Extracellular Vesicles a Marker of Advanced Lung Cancer?
Source: Cancers (Basel). 2020 Nov 20;12(11):3450. doi: 10.3390/cancers12113450 (PMC7699733; doi:10.3390/cancers12113450)
Supplement: Supplementary file 1 [file cancers-12-03450-s001.zip › cancers-984646-XML-suppl/cancers-984646-proofreading suppl.pdf]

cancers

MDPI

Supplementary Materials

Is the Proteome of Bronchoalveolar Lavage Extracellular Vesicles a Marker of Advanced Lung Cancer?

Ana Sofia Carvalho, Maria Carolina Strano Moraes, Chan Hyun Na, Ivo Fierro-Monti, Andreia Henriques, Sara Zahedi, Cristian Bodo, Erin M Tranfield, Ana Laura Sousa, Ana Farinho, Luís Vaz Rodrigues, Paula Pinto, Cristina Bárbara, Leonor Mota, Tiago Tavares de Abreu, Júlio Semedo, Susana Seixas, Prashant Kumar, Bruno Costa-Silva, Akhilesh Pandey and Rune Matthiesen

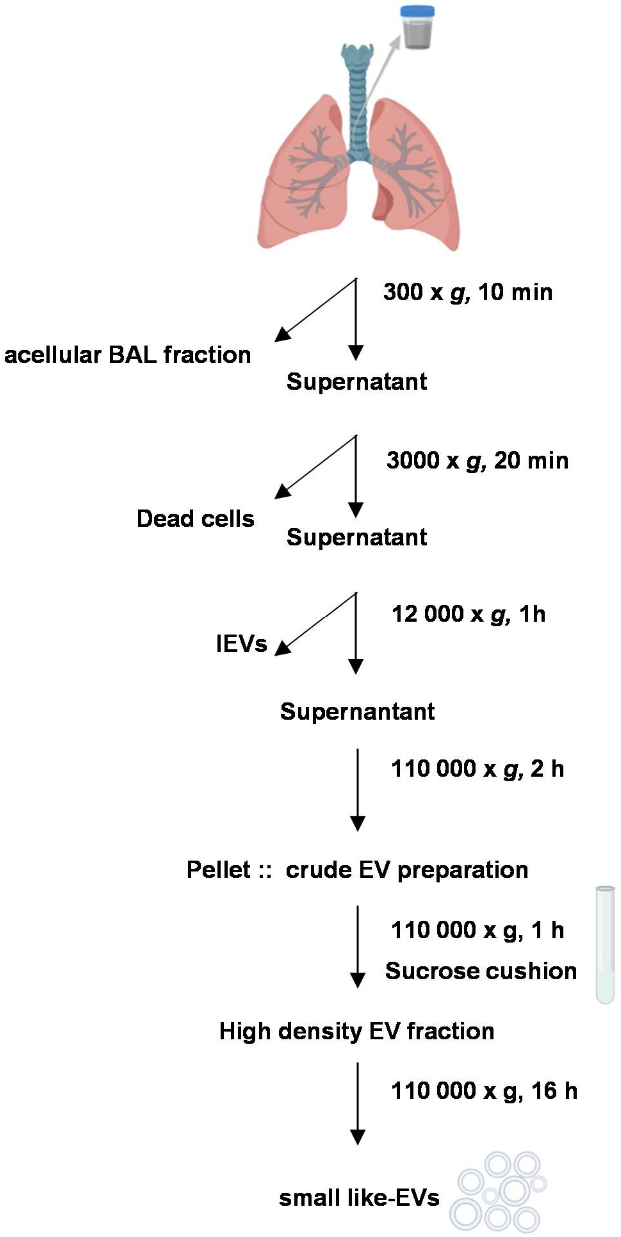

Figure S1. Schematic illustration of the small like-EVs and lEVs preparation.

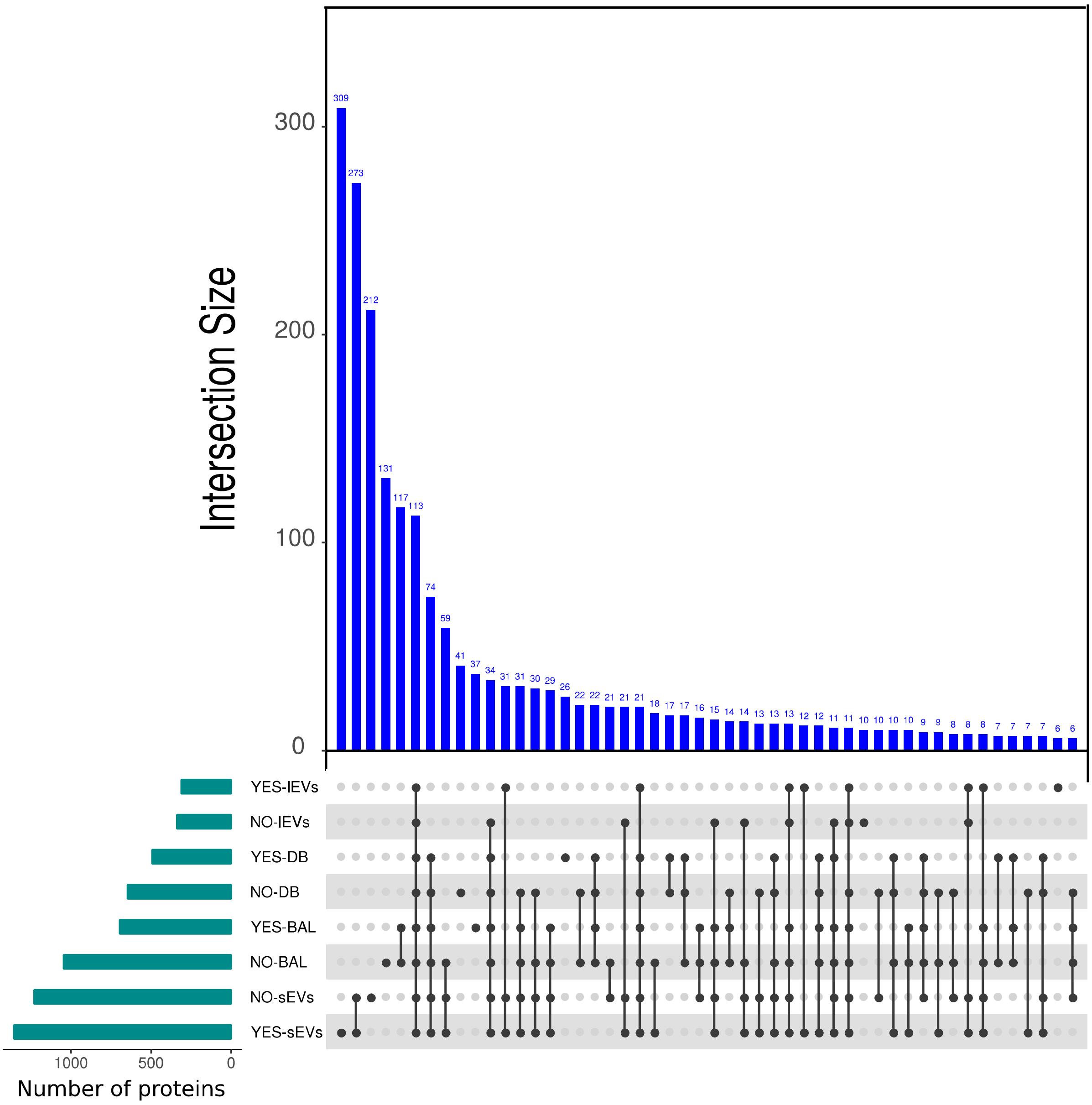

Figure S2. UpSet plot indicating the overlap between the identified proteins in each of the extracellular fractions for cancer (YES) and control (NO) samples. The barplot on the bottom left represents the total number of proteins identified in each sample/fraction type.

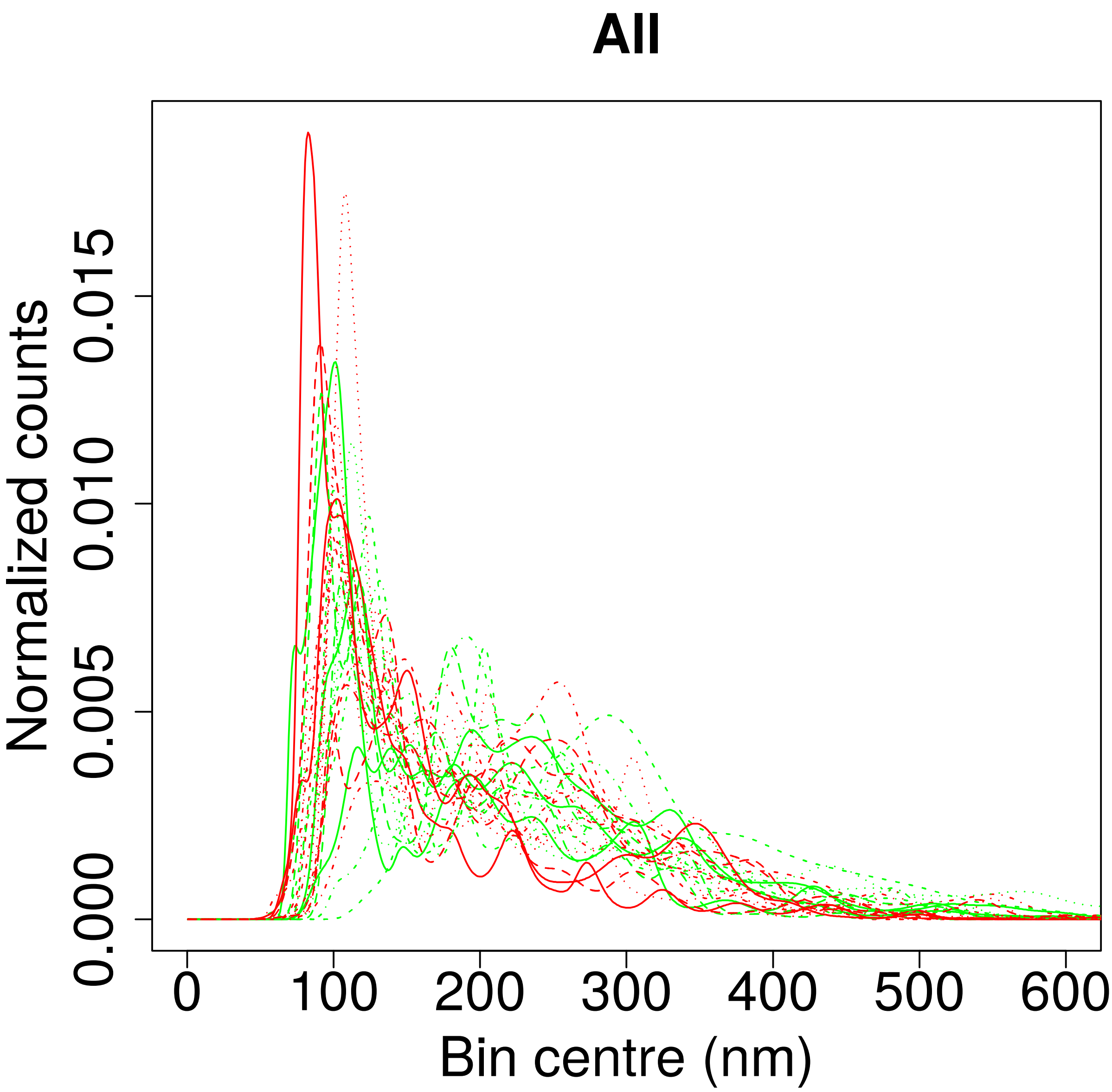

Figure S3. Normalised particle counts for all sEVs. Red indicate cancer patients and green controls.

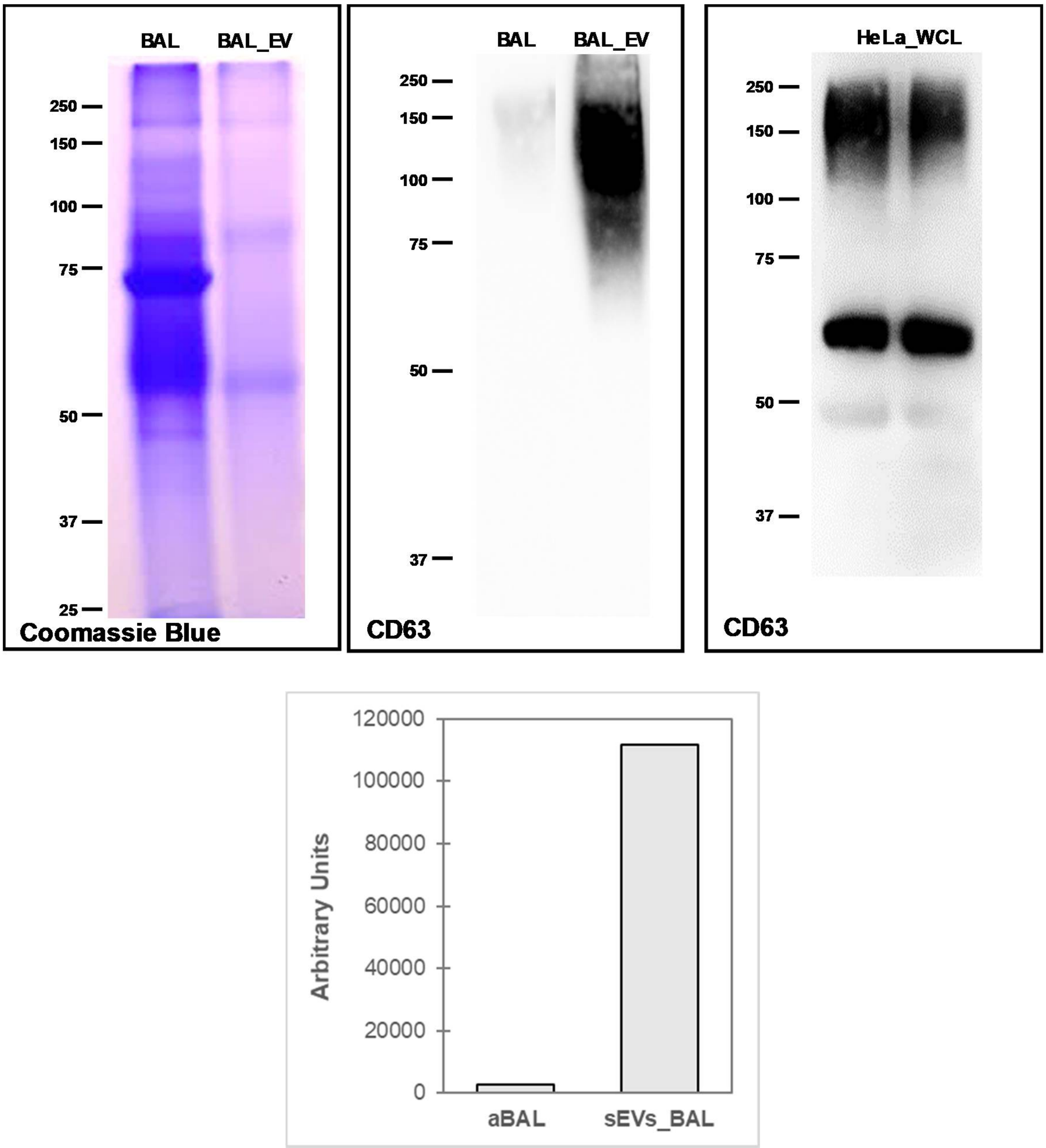

Figure S4. Western blot of CD63 enrichment in sEVs (upper panel) and densitometry quantitation (lower panel).

.

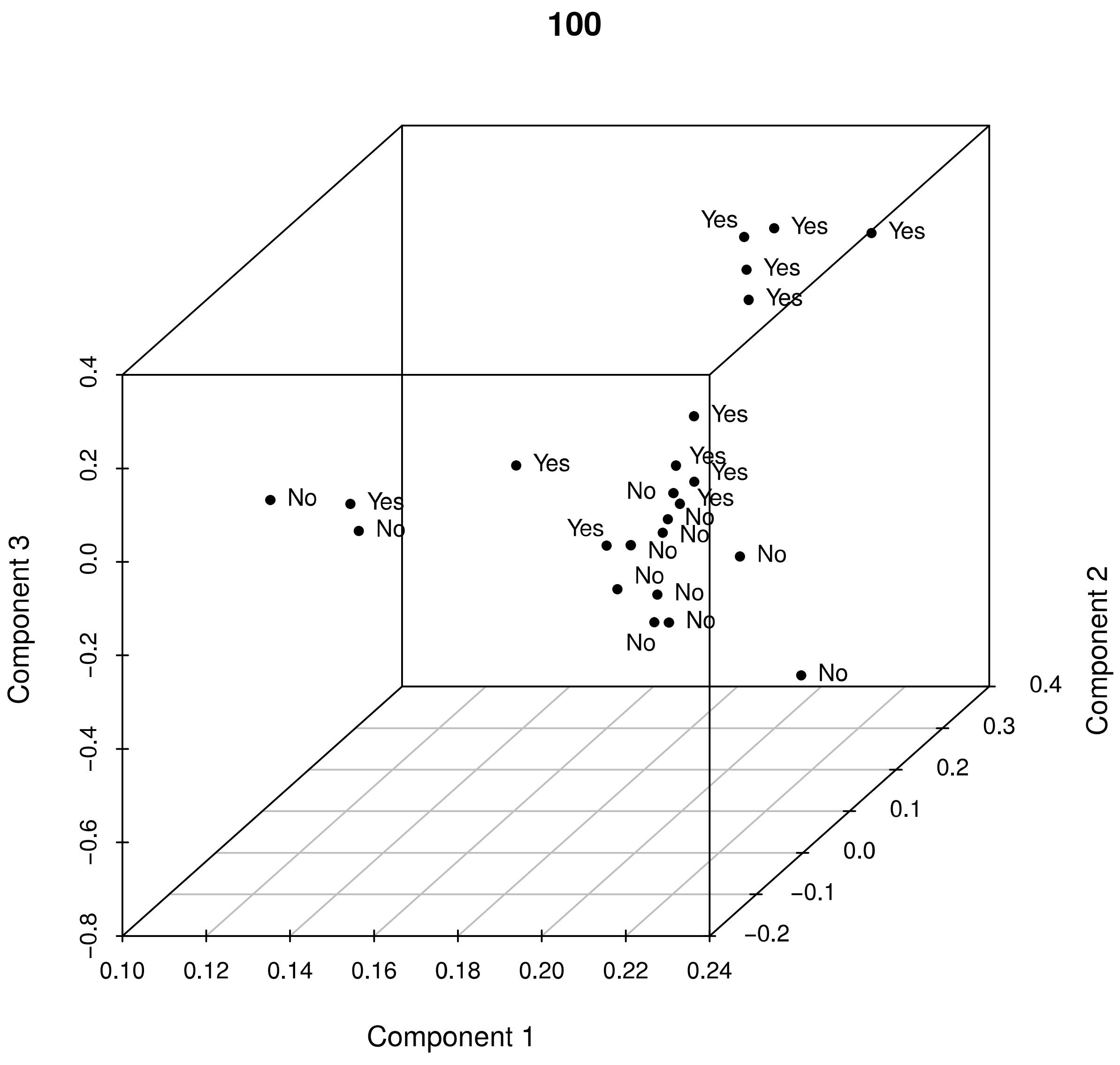

Figure S5. Principal component analysis using all log2 transformed raw expression values from MS on sEVs.

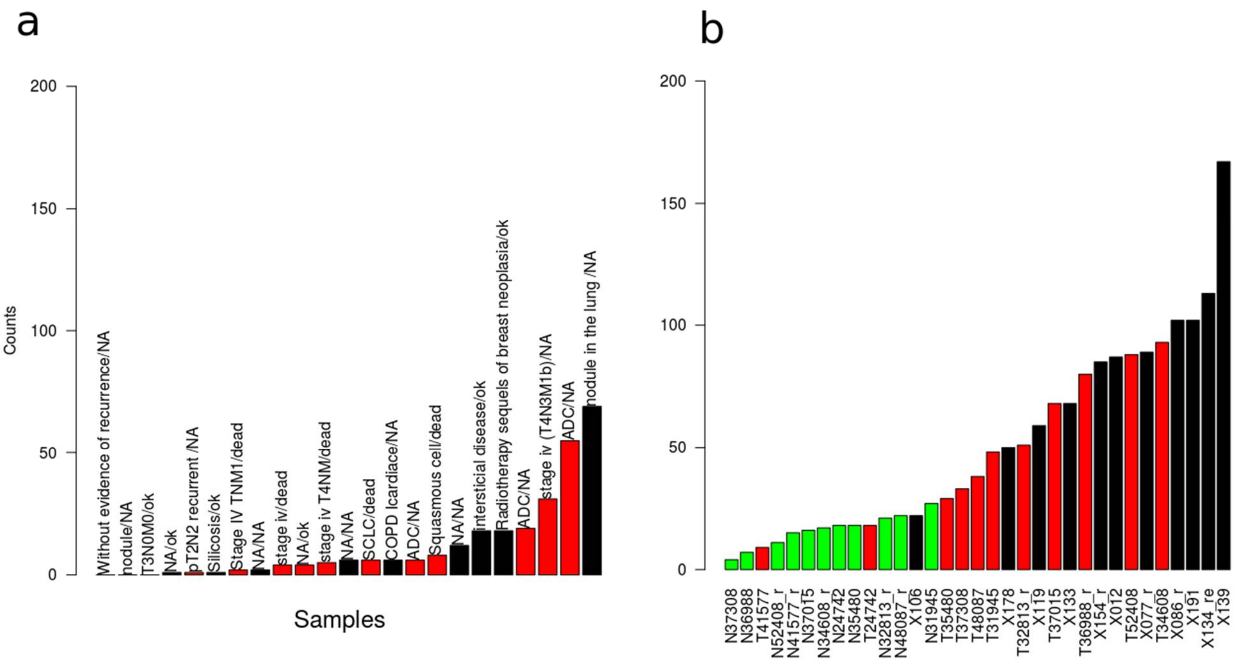

Figure S6. Unique proteins per sample identified in (a) acellular BAL and (b) tumor tissue (N: green normal tissue; T: red tumor tissue; and X: black mouse xenograft tissue).

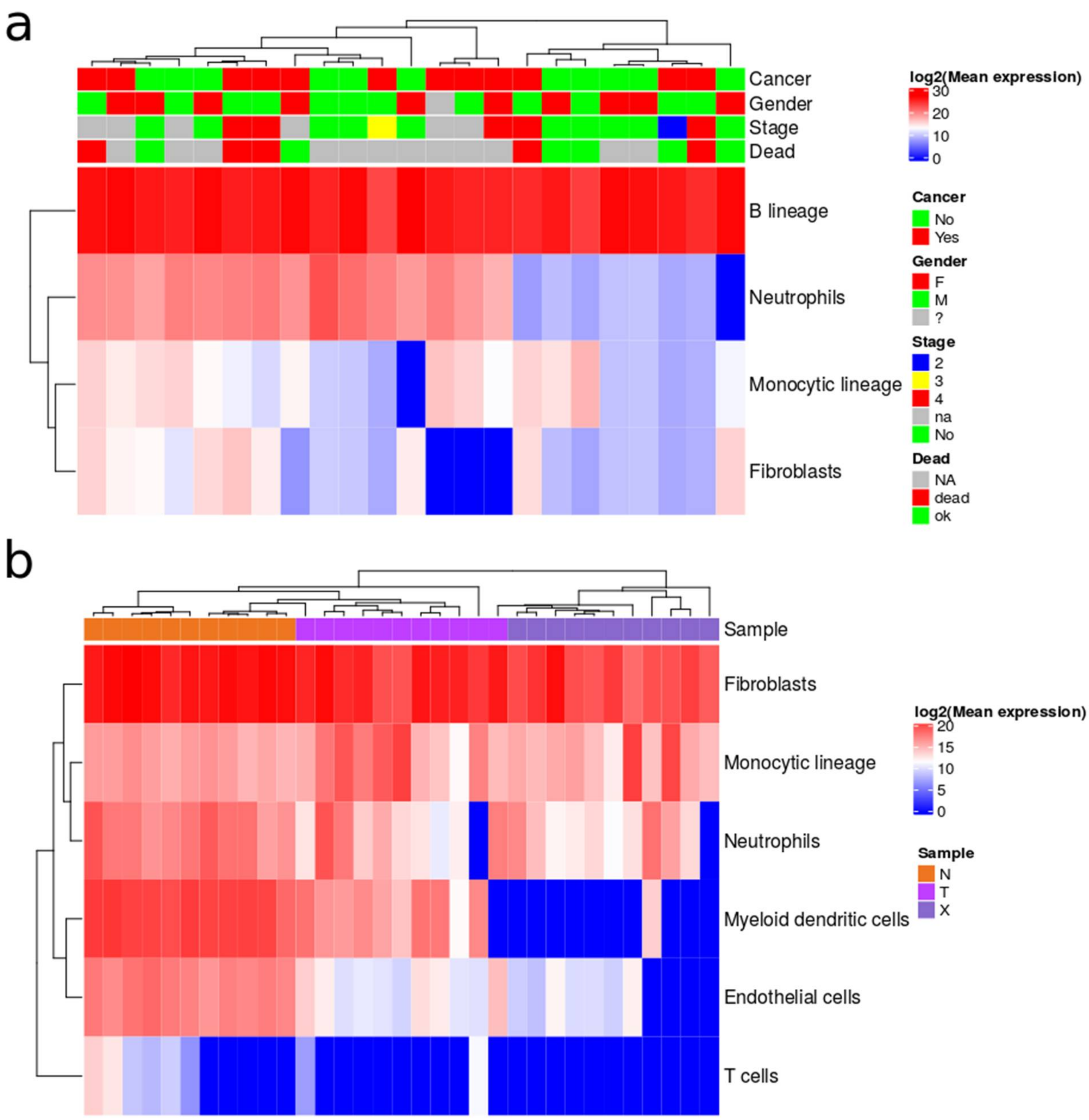

Figure S7. Average abundance scores of eight immune and two stromal cell populations protein markers: (a) based on acellular BAL proteomics; (b) normal (N), tumor (T) and xenotransplanted (X) tissue.
